# Timing and Spectrum of Neurological Complications After Flow Diverter Implantation for Intracranial Aneurysms

**DOI:** 10.3389/fneur.2021.590383

**Published:** 2021-04-20

**Authors:** Guillaume Charbonnier, Jean-Philippe Desilles, Simon Escalard, Benjamin Maier, Gabriele Ciccio, Stanislas Smajda, Robert Fahed, François Delvoye, Hocine Redjem, Raphaël Blanc, Michel Piotin, Mikael Mazighi

**Affiliations:** ^1^Interventional Neuroradiology, Besançon University Hospital, Besancon, France; ^2^Interventional Neuroradiology, Rothschild Foundation Hospital, Paris, France

**Keywords:** intracranial aneurysm, stents, platelet aggregation inhibitors, stroke, cerebral hemorrhage, subarachnoid hemorrhage

## Abstract

**Background and Purpose:** The aim of this study was to characterize neurological complications after flow diverter (FD) treatment on a long follow-up cohort and identify predictive factors associated with these complications.

**Methods:** This study was conducted on a monocentric cohort of patients treated for intracranial aneurysms by FD.

**Results:** Between September 2008 and July 2018, 413 patients were treated for 514 aneurysms: 18% of the patients presented with at least one neurological complication during a median follow-up of 446 days (IQR 186–1,210). Sixty-one patients presented with ischemic complications, 13 with hemorrhagic ones and 10 with compressive processes. Among 89 neurological complications 64.5% were peri-operative (occurring within the 30 days following the procedure) and 35.5% were delayed after 1 month.

**Conclusions:** Overall, neurological complications after FD implantation were overrepresented by cerebrovascular ischemic events occurring during the peri-operative period, but also in a delayed manner after 1 year. Long-term follow-up is relevant after aneurysm intervention using FD.

## Introduction

Flow-diverter stents (FD) are the latest generation of intracranial stents used to treat intracranial aneurysm, usually those with a wide neck or a fusiform morphology. Several studies reported complications after FD procedure (1–6), but mainly the most severe ones including disability and death. In fact, severe complications (such as aneurysm rupture and delayed hemorrhagic strokes) related to FD procedures were the source of intense debates in respect to the involved mechanism (7). Evidences on delayed neurological events, their type, timing, impact on therapeutic management and prognosis are lacking. The aim of this study was to characterize neurological complications after FD treatment on a long-follow-up cohort and identify predictive factors associated with these complications.

## Methods

### Study Design

This study was conducted on a monocentric cohort of patients included in 2 prospective studies, treated either for ruptured (Patients' Follow-up After Subarachnoid Hemorrhage Caused by Ruptured Intracranial Aneurysms - FUSAC -NCT02879175-) or unruptured intracranial aneurysms (Standardized Long-Term Follow-up of Patients After Endovascular Embolization of a Brain Aneurysm - ANENDOVASC -NCT02878967-) with FD. Our institutional ethics committee approved the two studies.

### Patients Characteristics

For each patient the following items were collected: date, age, sex, localization, aneurysm type and size, history of hemorrhage, previous endovascular treatment, modified Rankin Scale (mRS), neurological complication type, time to neurological complication, time to last follow-up. No ruptured aneurysm was treated using a flow diverter. Patients from the FUSAC study treated with FD, were patients with a history of ruptured aneurysm, but the aneurysm treated was either an associated unruptured aneurysm or a recurrence of the initial ruptured lesion.

Patients were treated in pre-operative period by aspirin and clopidogrel. Patients were then tested for clopidogrel resistance with P2Y12 Reaction Unit (PRU). If positive (>208), clopidogrel was changed for ticagrelor. The two antiplatelet agents were pursued 3 months and then only Aspirin was pursued for three additional months.

### Complications and Prognosis

Neurological complications were screened at each contact (following the standard of care in the department: 6 months, 1 year and every 2 years and additional visits if needed) and retrospectively reviewed, as well as, adjudicated by two neurologists (GC, MM). Complications were classified in four categories: ischemic, hemorrhagic, compressive and other. Among ischemic ones we identified: Acute Ischemic Stroke (AIS), and transient ischemic attack (TIA). Hemorrhagic strokes included: Parenchymal Hematoma (PH), and Subarachnoid Hemorrhage (SAH). Peri-operative complications were defined as events occurring within 30 days after procedure; and delayed complications, when events occurred afterwards. Neurological complications were characterized by a persistent deficit lasting more than 24 h. Retinal ischemia was classified as an AIS if a branch occlusion was seen. TIA were defined as transitory neurological symptoms (<1 h) without any evidence of brain ischemia. Good outcome was defined by a mRS 0–2 at last follow up.

### Statistical Analysis

An univariate logistic analysis was made with every possible factor for each endpoints (mRS 0–2; mRS 6; every neurological complication). The small amount of patient mRS 3–6 led to use least absolute shrinkage and selection operator (LASSO) which is a logistical regression with penalty in order to avoid overfitting. Variables which were selected by LASSO, were then computed for logistical regression without penalty and validated by bootstrap. Performance of the model was validated by area under the curve (AUC). Results with *p* < 0.05 were considered significant. Univariate analysis of complications type was done with non-parametric Fisher test. Each statistical analysis was made with R 3.4.3 with glmnet package for LASSO analysis.

## Results

Between September 2008 and July 2018, 413 patients with 514 aneurysms were treated. Population characteristics are listed in Table 1. Among the 447 procedures, we implanted 265 Pipeline (Medtronic, Dublin), 147 Silk (Balt, Montmorency), 20 FRED (Microvention, Aliso Viejo), 13 Surpass Streamline (Stryker, Kalamazoo), 1 p64 (Phenox, Bochum). One procedure failed and didn't lead to a stent implantation. Patients had a median follow-up of 446 days (IQR 186–1,210), with 64% with at least 1 year of follow-up. Eighteen percent of the patients presented with at least one neurological complication. Sixty-one patients presented with ischemic complications, 14 with hemorrhagic complications, 10 with compressive complications. One patient was diagnosed with aura-like spreading cortical depression characterized by progressive onset transitory symptoms, lasting for few minutes up to several hours (8) without any sign of ischemia on MRI. None of the hemorrhagic complication was due to aneurysm rupture after FD placement. Among these 73 patients with complications, 37 had complete or partial hemiparesis, four isolated aphasia or dysarthria, 17 visual loss, seven were early deceased and eight were asymptomatic. Among 61 patients with ischemic complications, 62% (38/61) remain disabled after 24 h.

**Table 1:** Characteristics of the population.

	***N***	**%**
Female	325	79
Age (mean, years) ± IQR	51.7 ± 8.7	
Previous SAH	62	15.0
Previous EVT % (number of aneurysms)	24.7% (127/514)	
**Aneurysm location**		
Anterior circulation	381	92.2
ICA	299	72.4
MCA	42	10.1
ACoA	24	5.8
Perical	16	3.9
Posterior circulation	32	7.8
Basilar	17	4.1
Vertebral	10	2.4
PCA	3	0.7
PICA	1	0.2
Superior cerebellar	1	0.2
**Aneurysm type**		
Blister	12	2.9
Dissecting	4	1.0
Fusiform	23	5.6
Size (mean, mm) ± IQR	8 ± 8.7	
Adjunctive coiling % (number of aneurysm)	19.8% (102/514)	
Number of FD (mean ± IQR)	1.19 ± 1.0	
**Outcomes**		
Patients with neurological complication(s)	74	17.9
mRS 0–2	377	91.5
mRS 6	13	3.2

*SAH, subarachnoid hemorrhage; EVT, endovascular treatment; ICA, Internal Carotid Artery; MCA, Middle Cerebral Artery; ACoA, Anterior Communicating artery; Perical, pericallosal artery; PCA, Posterior Communicating Artery; PICA, Posterior inferior cerebellar artery*.

### Factors Associated With Neurological Complications and Mortality

In univariate and multivariate analyses, neurological complications (all types) were associated with middle cerebral artery (MCA), posterior circulation aneurysm locations, and aneurysm size. Good outcome was associated with female sex, young age, internal carotid artery (ICA) aneurysm location, whereas mortality was associated with posterior circulation location.

### Timing of Neurological Complications

**Figure 1:**
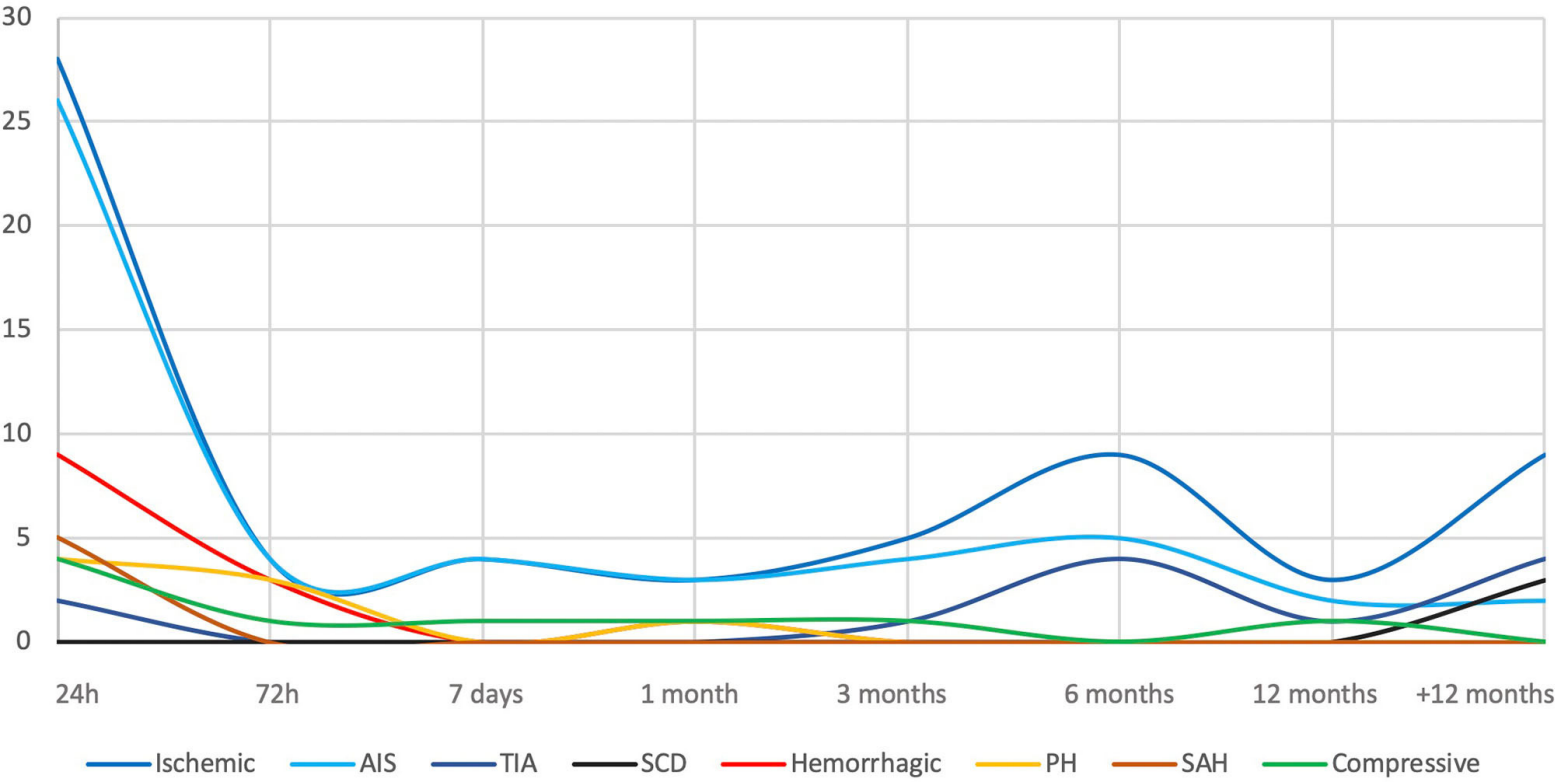
Complication types and timing. AIS, Acute Ischemic Stroke; TIA, Transient Ischemic Attack; SCD, Spreading Cortical Depression; PH, Parenchymal Hematoma; SAH, Subarachnoid Hemorrhage.

A total of 89 complications occurred, 64.5% were peri-operative and 35.5% were delayed. Neurological complications were predominantly ischemic either for peri-procedural or delayed ones. No hemorrhagic complication occurred after 30 days (Table 2, Figure 1). Of note, 8% (7/89 complications) presented a neurological complication within the 30 days following the discontinuation of an antiplatelet therapy (4 at 4th month, 1 month after clopidogrel stop; 3 at 7th month, 1 month after aspirin stop).

**Table 2:** Characteristics of the peri-procedural vs. delayed complications.

	**0–30 days**	**After 30 days**	***p***
Time to complication, mean, days	5	421	/
Ischemic all	64.4% (38/59)	90.0% (26/30)	0.4
AIS	59.3% (35/59)	53.3% (16/30)	0.85
TIA	5.1% (3)	33.3% (10/30)	0.0048
Compressive	11.9% (7/59)	10.0% (3/30)	1
Hemorrhagic all	23.7% (14/59)	0	0.009
PH	13.6% (8/59)	0	0.055
SAH	10.2% (6/59)	0	0.17
Other	0	3.3% (1/30)	0.34

*AIS, Acute Ischemic Stroke; TIA, Transient Ischemic Attack; PH, Parenchymal Hematoma; SAH, Subarachnoid Hemorrhage; Other refers to one patient with atypical regressive neurological deficits which were diagnosed as spreading cortical depression*.

## Discussion

In our study, 18% of patients treated with FD presented neurological complications. These complications were mainly ischemic events occurring within the first month (45% occurring after 3 days), but more than 35% occurred later than 1 year. Patients treated for large MCA, posterior circulation aneurysms were at higher risk of neurological complication. In addition, mortality was associated with posterior circulation locations. Despite these complication rates, 92% of the patients were functionally independent (mRS 0–2) within a median follow-up of 446 days.

Our series reports higher rates of complications in comparison to previous published ones (3, 9, 10). In the INTREPED study (3), which focused on AIS after FD implantation, the rate of ischemic events reached 4.5%. The majority (72.2%) of AIS occurred within 30 days, and the fusiform morphology of the aneurysms was the only variable associated with AIS (OR, 2.74; 95% CI: 1.11–6.75; *P* = 0.03). More recently, within a follow-up of 12 months, the prospective DIVERSION observational study (11) reported an event-free survival rate of 75.7%, with a 5.9% rate of permanent-related serious events and 14% of AIS out of 408 FD implantation. In the latter study, hypertension, diabetes and larger aneurysms were associated with neurological deficit occurrence. None of the previous studies reported neurological complications after 12 months. The discrepancy in the observed complication rate with our series, could be explained by a systematic follow-up consult at 6 months, 1, 3, and 5 years follow-up, that could have led to the detection of a higher complication rate. Also we treated 17 basilar artery aneurysms which are known to be associated with higher complications. Association between aneurysms location and neurological complications have been published and is consistent with our results regarding higher risk for MCA (12) and posterior circulation (13), possibly in relation to higher rate of perforators in these locations.

**Figure 2:**
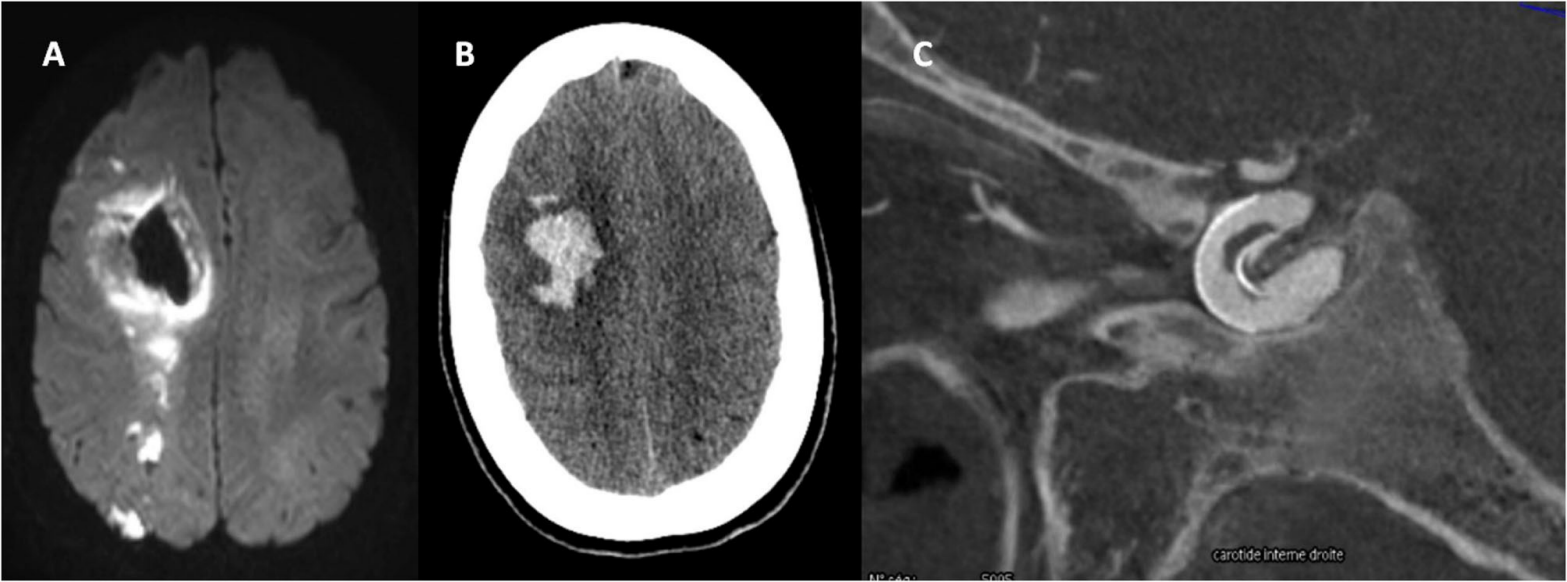
Patient who presented with a neurological deficit 4 days after the procedure. **(A)** Diffusion hypersignals in favor of carotid ischemic stroke. **(B)** Co-localization of the PH and AIS lesions supporting the ischemic mechanism. **(C)** Intra-stent thrombosis supporting ischemic mechanism of PH.

In the present study, TIAs were more frequently delayed and hemorrhages were observed only as peri-procedural events. AIS and TIA may be related to in-stent thrombosis with either reduced flow or emboli in the parent artery territory, whereas SAH were consecutive to per-procedural perforation. PH mechanism have been widely discussed considering adverse events consecutive to the use of antiplatelet agents (i.e., hemorrhagic worsening of a vessel perforation), or hyperperfusion syndrome after FD (1). In the present cohort several imaging patterns, including MRI imaging suggest the occurrence of AIS hemorrhagic transformations for PH mechanism. Figure 2 illustrates the case of a patient who presented with a neurological deficit 4 days after the procedure. Diffusion-weighted hypersignals co-localized with PH were supporting the ischemic mechanism, with in-stent thrombosis documented in the follow-up angiography. In respect to the 12 patients experiencing a TIA, a therapeutic change was undertaken mostly by the reintroduction of the antiplatelet therapy. Afterwards, none of the 12 patients presented a recurrent stroke. Of note, seven complications occurred within the first month following an antiplatelet agent cessation. These cases raise the question of the antiplatelet therapy optimal duration for FD. This duration and the nature of antiplatelet therapy remains unclear according to a recent meta-analysis (14), which describes a 6.5% of ischemic complications vs. 3.9% of hemorrhagic complications after FD procedure. A prolonged use of antiplatelet therapy may lower ischemic complications but increase the hemorrhagic risk. Two other studies did systematic reviews and found similar rates, with an oassociation between ischemic complication and a duration of clopidogrel inferior to 6 months (15, 16). There is therefore a need to identify the population at higher risk of ischemic events. Patients with large MCA or vertebrobasilar aneurysms are potentially the subgroup of patients requiring a prolonged antiplatelet therapy. Additional evidences are needed to identify the patients that may benefit from a prolonged anti-thrombotic therapy.

Our study has some limitations. Firstly, this is a retrospective analysis including heterogeneous patients with ruptured and unruptured aneurysms. We cannot affirm causal links between the observed associations and patient outcomes. In addition, although the median follow-up was over 1 year, we could have underestimate neurological complications occurring later.

The spectrum of neurological complications after FD procedures reveals a predominance of ischemic vascular events with hemorrhagic ones occurring only in the peri-procedural period. Long follow-up is relevant in this population since delayed complications may take place later than 1 year.

## Data Availability Statement

The raw data supporting the conclusions of this article will be made available by the authors, without undue reservation.

## Ethics Statement

The studies involving human participants were reviewed and approved by Comité de protection des personne Île de France III and Comité de protection des personne Île de France IV. Written informed consent for participation was not required for this study in accordance with the national legislation and the institutional requirements.

## Author Contributions

GCh and MM conceived the study. GCh, MM, and MP wrote the manuscript. GCh, MM, MP, J-PD, SE, BM, GCi, SS, RF, FD, HR, and RB collected the data. All authors contributed to the article and approved the submitted version.

## Conflict of Interest

The authors declare that the research was conducted in the absence of any commercial or financial relationships that could be construed as a potential conflict of interest.
